# Assessing the effectiveness of mindfulness-based programs on mental health during pregnancy and early motherhood - a randomized control trial

**DOI:** 10.1186/s12884-019-2503-4

**Published:** 2019-10-10

**Authors:** Wan-Lin Pan, Chiung-Wen Chang, Shin-Ming Chen, Meei-Ling Gau

**Affiliations:** 1 Department of Nursing, National Taipei University of Nursing and Health Sciences, No. 365, Ming-Te Road, Peitou, Taipei, 11219 Taiwan; 2 Taiwan Adventist Hospital, No.424, Sec. 2, Bade Rd., Songshan District, Taipei, 10556 Taiwan; 3 Department of Nurse-Midwifery and Women Health, National Taipei University of Nursing and Health Sciences, No. 365, Ming-Te Road, Peitou, Taipei, 11219 Taiwan

**Keywords:** Pregnancy, Postpartum, Stress, Depression, Mindfulness

## Abstract

**Background:**

The process of entering motherhood is highly stressful for women, with 15–85% of new mothers experiencing postpartum blues or depression. This study was designed to evaluate the efficacy of a mindfulness-based childbirth and parenting program in improving psychological health during the postpartum period.

**Methods:**

This research was a randomized controlled trial with single blinding. Recruitment began after the participating hospital granted formal approval. A total of 74 women between 13 and 28-weeks gestation were allocated either to the intervention group or to the comparison group. The intervention program included a series of eight, 3-h classes held once weekly and 1 day of 7-h silent meditation. Psychological health was assessed at baseline and 3-months postpartum.

**Results:**

Significant differences in stress and depression were observed in both groups over time. Stress scores and depression scores were significantly better in the intervention group than in the comparison group at 3-months postpartum (F = 7.19, *p* = .009 and F = 7.36, *p* = .008, respectively). No significant difference between the groups was identified for mindfulness scores at 3-months postpartum.

**Conclusions:**

The intervention program effectively reduced postpartum self-perceived stress and depression, suggesting that this program provides acceptable and long-term benefits to women during pregnancy and the postpartum period. The teaching and practice of mindfulness meditation and parenting education during pregnancy may help reduce stress and depression in pregnant women as they transition into parenthood.

**Trial registration:**

The ClinicalTrials.gov identifier for this study is: NCT03185910. The study was retrospectively registered on 14 June 2017.

## Background

Becoming a mother is often a stressful transition [1], involving long-term processes that qualitatively reorganize both inner thoughts and external behaviors [2]. This transition may be fraught with difficulties and additional stresses that render new mothers more vulnerable to postpartum depression [3].

An increasing body of evidence suggests that perinatal stress and depression are symbolic expressions of powerlessness that negatively affect new mothers’ intimate and social lives (e.g., poor attachment with their babies and reduced interpersonal interactions) and have both short- and long-term negative effects on mothers and newborns [4] (e.g., increased incidence of preterm birth, fetal growth retardation, and low Apgar scores) [5, 6].

These adverse outcomes are associated with increased risks to the neurobehavioral and cognitive development of babies and children [7]. Depression has received increased attention in recent years. The global prevalence of postpartum depression (PPD) is currently estimated at 17.7% (95% confidence interval: 16.6–18.8%) [8]. As women are often required to continue shouldering heavy work, social, and family demands while caring for their newborn child, the mental health of postpartum women deserves particular attention.

A significant body of peer-reviewed and published evidence supports that stress inventories are able to predict depression [9]. Therefore, reducing perinatal psychological distress (stress, depression, and anxiety) during pregnancy and the first-year postpartum should be a crucial public-health goal [10]. Although pregnancy is often presented in the literature as a stressful period, some women welcome pregnancy as a challenge. Pregnancy is also known to be an opportune time for suggesting health interventions [11]. However, the longitudinal effect of psychological antenatal education interventions during the postpartum period have rarely been evaluated. In Taiwan, most hospitals offer a routine, several-hour childbirth education course on a once-monthly basis, with no restriction on the number of women or couples attending the class. These courses adopt a unidirectional teaching strategy, and interactions between instructors and participants or among participants is not encouraged. Course content addresses the physiological changes during pregnancy and self-care for physical discomfort, preparing for labor, comfort measures during labor, epidural anesthesia and cesarean section-related knowledge. However, these courses typically do not teach strategies for self-managing psychological health issues during the perinatal period.

The psychological health of pregnant populations has improved since mindfulness-based interventions (MBIs) were introduced in the 1990s. Evidence now supports MBIs as an effective approach to reducing psychological stress in Europe and North America [12] and as effective in reducing stress [13–16], anxiety [13–15, 17–19], and depression [14, 16, 18–21] during pregnancy. Furthermore, some researchers have found that the effects of MBIs persist through 4–6 weeks postpartum [14, 15, 20–22]. Nevertheless, few studies have investigated the duration of mindfulness beyond six-weeks postpartum.

Although the benefits of mindfulness-based interventions during pregnancy and postpartum have been previously demonstrated, the impact of these interventions on mothers during early parenthood is unclear. Therefore, the purpose of this study was to assess the efficacy of an MBI on the long-term psychological health of women during pregnancy and into early motherhood.

## Methods

A randomized, controlled trial was conducted to measure the effects of the mindfulness-based childbirth and parenting (MBCP) program (intervention group) compared to the hospital’s routine childbirth education (comparison group). In addition, both groups received routine antenatal care from the research hospital.

### Procedures

The required sample size was calculated using statistical power analysis. The results of a previous MBCP study [21] show mean and standard deviation values of 8.3 (SD = 6.1) and 12.9 (SD = 9.1) for the experimental group and control groups for the depression scale, and a statistical power of 0.8 was used to reject a null effect at the 0.05 level of significance. A minimum sample size of 36 for each group was calculated and, after taking into account a possible attrition rate of 40%, a target sample size of 104 participants was set [23].

The randomization sequence was generated on a computer using a random block size that assigned participants to either the MBCP group or the comparison group in a 1:1 ratio [24]. Participants were randomized, envelopes were packaged, and group numbers were hidden in successively numbered, opaque sealed envelopes. The study was single-blinded only, as the researchers explained the meaning of mindfulness to each participant. The research assistant worked with staff at the target hospital to identify women who met the inclusion criteria, and these women were approached during hospital visits while in the waiting rooms. Those who met the inclusion criteria were invited to participate.

**Fig. 1:**
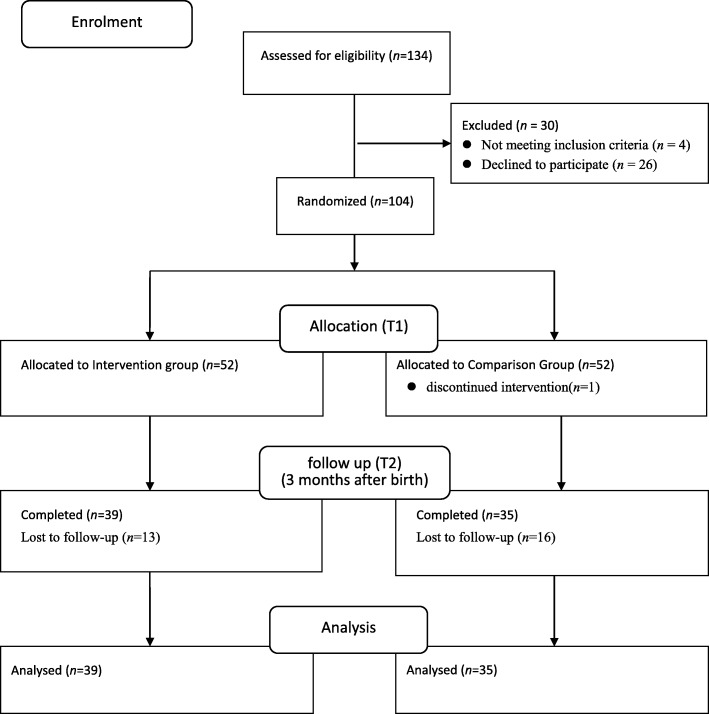
CONSORT diagram of all participants at T1 and T2

This study was conducted at a regional hospital that provides regular healthcare and maternity services in northern Taiwan and that delivers approximately 2000 births yearly. Recruitment was carried out in the hospital’s antenatal clinics. Patients who met the following criteria were invited to participate: singleton pregnancy between 13 and 28 weeks, at least 20 years of age, able to read and speak Chinese, and interested in attending the prenatal class. Otherwise eligible patients were excluded if they had a history of mental illness or known inability to attend more than six prenatal class sessions. The final sample size for this study was 74 participants, with 35 in the comparison group and 39 in the intervention group. The consolidated standards of reporting trials (CONSORT) participant flow diagram is presented in Fig. 1. The mean attendance of participants was 7.18 out of 8 sessions.

### Intervention and comparison group programs

The intervention group received the MBCP intervention in a group setting once per week for eight consecutive weeks. The first author, who had previously completed MBCP and MBSR teacher training, was responsible for teaching the intervention group intervention course. The MBCP program treatment group consisted of 8–15 participants who met for one three-hour session per week for 8 weeks. The participants were encouraged to complete the mindfulness exercises during sessions and practice 6 days each week for 30 min each day at home using the provided recorded audio. The intervention method is described in detail in a previous publication [16]. The groups met on separate Sunday afternoons from 13:00 to 16:00, and the research period was from February 2nd, 2016 to August 1st, 2017.

The session involved teaching participants how to monitor their sensory and emotional states and cognitive processes, deepen their sensory self-awareness, and become more mindful of the process of labor and parenting. The MBCP techniques were based on Nancy Bardack’s “Mindful birthing: training the mind, body, and heart for childbirth and beyond”. The participants were taught to use formal and informal mindfulness to cope with the stressful events of pregnancy, childbirth, and the postpartum period. The formal mindfulness practices covered: body scan, mindful yoga, being with the ice, three-minute breathing space, and seated meditation. The informal mindfulness method involved the participants noticing their experience from moment to moment and concentrating their attention on one thing as many times as possible throughout the day [25].

The comparison group received a standard presentation on physiological and psychological information and practice skills during pregnancy and after childbirth from two nurse-midwives at the participating hospital. The comparison group received no information or instructions on mindfulness concepts or practices. The presentation lasted 4 h and was delivered at the hospital.

### Measures

The demographic information that was collected for this study included: age, gestational age at enrollment in the study, education, marital status, religiosity, employment status during pregnancy, income, pregnancy intention, and parity. The primary outcome measure, perceived stress, was measured using the Perceived Stress Scale (PSS-10) [26], a 10-item, 5-point Likert scale scored from 0 (never) to 4 (very often), with higher scores corresponding to higher perceived stress. This scale earned a Cronbach’s alpha of .87 for this study.

Secondary outcome measures included changes in the Edinburgh Postnatal Depression Scale (EPDS) [27] and Five Facet Mindfulness Questionnaire (FFMQ) [28] scores. The EPDS is a 10-item, 4-point Likert scale scored from 0 (No, not at all) to 3 (Yes, most of the time) that is designed to identify patients at risk for perinatal depression, with higher scores corresponding to higher perceived depression. The FFMQ is a 39-items scale, 5-point Likert scale scored from 1 (never or very rarely true) to 4 (very often or always true). The developers of the FFMQ conducted exploratory factor and confirmatory factor analyses on all of the items using many mindfulness measures to generate the five subscales of the FFMQ. The FFMQ subscales are: observing, describing, acting with awareness, non-judging of inner experience, and non-reactivity. The reported Cronbach’s alpha reliability of the scales in this study was .84 for the EPDS and .87 for the FFMQ.

The questionnaire was administered at two time points: immediately after informed consent was given during mid-pregnancy (T1) and 3 months after birth (T2). Approximately 15 min were required to complete the questionnaire.

### Statistical analysis

Data were analyzed using the Statistical Package for Social Sciences (SPSS) 22.0. Descriptive statistics were used to analyze frequency distributions, percentages, means, and standard deviations. Statistical significance was defined as *p* ≤ .05. An independent *t* test was used to compare the continuous demographic and obstetric data, including age and gestational age at enrollment, between the experimental and comparison groups. A chi-square test was used to compare the categorical data in terms of the level of education, marital status, religiosity, employment status, income, pregnancy intention, and parity. To control for potentially confounding variables, analyses of covariance (ANCOVA) were used to compare changes in scale scores between the groups at 3-months postpartum.

### Results

The distributions of sociodemographic factors are shown in Table 1. Women (*n* = 134) were assessed for eligibility and 30 were excluded for reasons including fetal chromosomal abnormality (*n* = 1), previous miscarriage (*n* = 1), history of pregnancy-induced hypertension (*n* = 2), and declined for personal reasons (*n* = 26). A total of 104 participants returned the first questionnaire and were randomized into the two groups, with 52 allocated to the intervention group and 52 allocated to the comparison group. At T2 (3-months postpartum), 74 participants (71.2%) returned the questionnaire, 29 were lost to follow-up, and 1 discontinued the intervention. Of those who returned the questionnaire, 39 (75%) were in the intervention group and 35 (67.3%) were in the comparison group.

**Table 1:** Comparison of participants who completed the questionnaire at T2 with those who did not

Participant characteristic	Completed questionnaire (*N* = 74)	Lost to follow-up (*N* = 30)	*p* value
Age: *M* (*SD*)	32.8 ± 3.9	33.8 ± 3.9	.219^c^
Week of gestational age at enrollment	20.7 ± 4.8	22.8 ± 5.2	.052^c^
Level of education			
Junior college or below	9	4	.551^a^
University or above	65	26	
Marital Status			
Married	73	27	.130^b^
Not married	1	3	
Religiosity			
Religious	36	10	.071^a^
Not religious	38	20	
Employment status◎			
Employed	60	24	.843^a^
Unemployed	14	5	
Income◎			
Less than US$1500	11	4	.982^a^
US$1500–US$2999	22	9	
More than US$2999	39	16	
Pregnancy intention			.074^a^
Intentional	53	16	
Unintentional	21	14	
Parity			.199^a^
No prior births	68	25	
1 or more prior births	6	5	

^a^Chi-Square test; ^b^Fisher’s Exact Test; ^c^Independent t test; ◎Numbers may sum to less than 96 because of missing data; Significant at the *p* ≤ .05

There were no significant differences between the participants who completed the questionnaire at T2 and those who did not in terms of age, gestational age, level of education, marital status, religiosity, income, employment status, income, pregnancy intention, or parity. Changes in scores for stress (PSS), depression (EPDS), and mindfulness (FFMQ) were calculated for both groups, with results shown in Table 2. Differences between the participants in the two groups were not significant at baseline. At 3-months postpartum, the PSS and EPDS scores had decreased and the FFMQ scores had increased by 9.29 from the baseline in the intervention group, while the comparison group had changed only minimally from the baseline for all scores.

**Table 2:** The pretest and post-test of the PSS, EPDS, and FFMQ

Variables	Intervention group (*n* = 39)					Comparison Group (*n* = 35)				
	Pretest		Post-test		Change	Pretest		Post-test		Change
	*Mean*	*SD*	*Mean*	*SD*		*Mean*	*SD*	*Mean*	*SD*	
PSS	15.41	5.74	11.64	6.13	−3.77	13.80	6.00	14.29	5.23	0.49
EPDS	9.49	3.95	6.51	4.51	−2.98	8.74	4.46	8.77	3.41	0.03
FFMQ	130.38	14.16	139.67	16.88	9.29	136.06	14.58	137.66	16.39	1.60
Observing	29.33	4.68	30.90	5.20	1.57	29.29	4.27	29.66	6.72	0.37
Describing	26.33	5.10	28.92	6.15	2.59	28.34	5.17	29.00	5.46	0.66
Awareness	28.00	4.01	28.87	4.56	0.87	29.54	3.40	29.91	4.34	0.37
Non-judging	23.54	4.10	25.85	5.07	2.31	26.00	4.98	27.06	3.61	1.06
Non-reactivity	23.18	3.83	25.13	4.27	1.95	22.89	3.39	22.03	4.51	−0.86

*PSS* Perceived Stress Scale, *EPDS* Edinburgh Postnatal Depression Scale, *FFMQ* Five Facet Mindfulness Questionnaire

PSS scores in the 14–26 range indicate a moderate level of stress, while those in the 27–40 range indicate a high level of stress [26]. The mean change in PSS score of − 3.77 (15.41 to 11.64) observed in the intervention group is thus clinically meaningful. Moreover, significantly more participants in the intervention group (*n* = 27/39*,* 69%) achieved significant decreases in stress than in the comparison group (*n* = 16/35, 46%). Similarly, using an EPDS score of 13 as the cutoff, 10 in the intervention group and 8 in the comparison group identified as having depression at T1, while 2 in the intervention group and 6 in the comparison group identified as having depression at T2, showing a significant improvement in depression in the intervention group.

Partial eta-squared, a formula widely used to measure effect size in education research [29], was used as the effect-size measure. Effect size estimates for analysis of variance were determined using partial ƞ^2^, with .01 defined as a small effect size, .06 defined as a medium effect size, and .14 defined a large effect size [30].

After adjusting for baseline scores, a more significant between-group difference was found for stress (*F* (1,71) =7.19, *p* = .009; medium effect size, partial ƞ^2^ = .09) and depression (*F* (1,71) = 7.36, *p* = .008; medium effect size, partial, partial ƞ^2^ = .09) in the intervention group than in the comparison group, with the intervention group reporting significantly lower levels of stress and depression at T2 than their comparison group peers. Nonsignificant results were found for the FFMQ subscales (*F* (1,71) = 3.62, *p* = .06; small effect size, partial ƞ^2^ = .05) with the exception of the non-reactivity subscale, which was higher in the intervention group than in the comparison group (*p* = .003; Table 3).

**Table 3:** The effects of the interventions on postpartum stress, depression, and mindfulness (*N* = 74)

Variables	B	*F* value	*p*	Partial η^2^	Power
PSS	−3.30	7.20	.009	.09	.75
EPDS	−2.46	7.36	.008	.09	.76
FFMQ	6.02	3.62	.061	.05	.47
Observing^a^	1.23	.81	.372	.01	.14
Describing^a^	.93	.56	.458	.008	.11
Awareness^a^	−.30	.09	.763	.001	.06
Non-judging^a^	−.91	.72	.398	.01	.14
Non-reactivity^a^	3.01	9.17	.003	.11	.85

^a^Comparison group as a reference group; Significant at the *p* ≤ 0.05

## Discussion

This study aimed to determine the effects of an 8-week mindfulness training intervention on a sample of pregnant women using a comparison group as the control. A total of 74 women completed the trial, with results empirically supporting most of the hypotheses. There are no significant between-group differences in psychological measures and demographic characteristics were found at T1. The intervention group realized a significantly greater decline in self-reported stress and depression than in the comparison (standard care) group. The mindfulness skills learned during the intervention had long-term mediating effects on the intervention-group participants, who maintained a low level of stress and depression at least to T2. In addition, moderate effect sizes (η^2^ > .06) were observed. The mindfulness intervention may best be administered during both the prenatal and postnatal periods in order to sustain its beneficial effects [18].

The closest comparable study is a pilot RCT conducted by Vieten and Astin (2008), which randomly assigned 31 healthy and pregnant women into two groups, with the intervention group receiving 8 weeks of Mindful Motherhood program training. Although significant differences in anxiety and negative affect were found in the post-test results, no between-group differences in these two variables were detected at 3 months after completion of the intervention. Although another study that focused on a new online course obtained results similar to those of this study, the 8-week postnatal follow-up response rate in that study was excessively low, at only 25.9% [31]. In addition, a randomized trial study that focused on pregnant women with psychological diseases found that 86 pregnant women with histories of depression reported significantly better depressive outcomes at 1 and 6-months postpartum than participants receiving treatment as usual [32]. This difference in findings may be due to the differences in sample populations and sample sizes used. The small to medium effect sizes in depression, anxiety, and stress are necessary factors to be considered for mindfulness in pre- to post-analysis research [12]. This study considered whether the identified differences in mental health persisted into the longer postpartum period.

Although the findings are consistent with previous research that has shown mindfulness to be effective at improving psychological well-being in different perinatal populations [14, 20, 21], the measurements in most of this research were conducted at 4–6 weeks postpartum. Few studies in the literature have assessed the effects of prenatal interventions at three-months postpartum (T2) or later. Thus, the longer-term duration of intervention effectivness is an area that deserves further exploration in future studies.

In terms of mindfulness, a previous study that used a one-group, pre–post experimental design with 18 subjects found that postnatal mindfulness increased between 3 to 12-weeks postpartum [22]. Another study that used a pilot RCT design (*n* = 47) found that participants in both groups experienced increased mindfulness at 6-weeks postpartum [15]. In this study, with the exception of the non-reactivity subscale of the FFMQ, the between-group differences did not significantly differ at T2. However, the mean FFMQ score for the intervention group increased by 9.29 between T1 and T2, while that for the comparison group increased by only 1.60. A longitudinal study conducted over a longer time period and using a larger sample should be conducted to verify and further explore the observed positive effect on mindfulness.

Two factors may underlie the ameliorating effect of mindfulness on stress-related factors. First, recent research has found that practicing mindfulness reduces depression and increases happiness in practitioners. MBIs are designed to increase psychological flexibility [14], which is a fundamental aspect of health [33]. Psychological flexibility encompasses a wide range of human capabilities and is thus adaptable to a variety of situational needs, helping practitioners keep important aspects of their life in balance and promoting consistent behaviors with awareness and openness [33], which may significantly benefit psychological health during prenatal and postnatal periods [20–22]. Second, the intervention group may have benefitted from a ‘group therapy’ effect attributable to the regular attention of researchers and study staff in a therapeutic environment. By contrast, the comparison group received only 4 h of total interaction time with the researchers. Therefore, holding weekly sessions may improve the effect of interventions [34].

Some limitations in the design of this study must be considered. First, this study had a high rate of attrition. A loss of 20% or greater indicates a significant risk of bias [35], which may influence the data supporting the potential benefits of mindfulness in the perinatal period. Second, this was a single-blind randomized controlled trial and the researchers were aware of the group assignments of the participants. Third, participants in the intervention group were encouraged to listen to the recorded instructions and to practice the MBCP at home. However, the actual time spent in home practice was not tracked. Future research examining the practice time of participants is needed in order to better understand the effects of mindfulness on postpartum women.

## Conclusions

The findings of this study highlight the potential efficacy of an 8-week MBCP intervention in improving stress and depression in postpartum women. Women in Chinese cultural settings typically stay at home for about 30 days after giving birth in order to recover from the birthing experience and return to health. This is a practice known as “doing the month” [36]. Thus, implementing a long-term, effective mental health course during pregnancy is especially important in this population. Mindfulness programs may be an effective approach to enhancing the mental health of women during pregnancy and the postpartum period. As mindfulness must be practiced before giving birth, including mindfulness courses and related practical exercises in prenatal education is recommended in order to promote well-being and reduce stress and depression in the postpartum period.

## Data Availability

The datasets used and analyzed in the current study are available from the corresponding author on reasonable request.

## Abbreviations

ANCOVA: Analyses of covariance
EPDS: Edinburgh Postnatal Depression Scale
FFMQ: Five Facet Mindfulness Questionnaire
MBCP: Mindfulness Based Childbirth and Parenting
MBIs: Mindfulness Based Interventions
PPD: Postpartum depression
PSS: Perceived Stress Scale
RCT: Randomized Controlled Trial
SPSS: Statistical Package for Social Sciences
T1: Time 1 (baseline/pre-intervention/pretest)
T2: Time 2 (3 months after birth/post-test)
